# Clinicians’ views and experiences of offering two alternative consent pathways for participation in a preterm intrapartum trial: a qualitative study

**DOI:** 10.1186/s13063-017-1940-5

**Published:** 2017-04-26

**Authors:** Celine Y. Chhoa, Alexandra Sawyer, Susan Ayers, Angela Pushpa-Rajah, Lelia Duley

**Affiliations:** 1 Centre for Maternal and Child Health Research, School of Health Sciences, City University London, London, EC1R 1UW UK; 2 Centre for Health Research, School of Health Sciences, University of Brighton, Falmer, BN1 9PH UK; 3 Department of Dermatology, Guy’s Hospital, London, SE1 9RT UK; 4 Nottingham Clinical Trials Unit, University of Nottingham, Nottingham, NG7 2UH UK

**Keywords:** Clinical trials, Ethics, Consent, Oral assent, Preterm birth

## Abstract

**Background:**

The Cord Pilot Trial compared alternative policies for timing of cord clamping at very preterm birth at eight UK hospitals. Preterm birth can be rapid and unexpected, allowing little time for the usual consent process. Therefore, in addition to the usual procedure for written consent, a two-stage pathway for consent for use when birth was imminent was developed. The aims of this study were to explore clinicians’ views and experiences of offering two consent pathways for recruitment to a randomised trial of timing of cord clamping at very preterm birth.

**Methods:**

This was a qualitative study using semi-structured interviews. Clinicians from eight hospitals in the UK who had been involved in offering consent to the Cord Pilot Trial were invited to take part in an interview. Clinicians were interviewed in person or by telephone. Interviews were analysed using inductive systematic thematic analysis.

**Results:**

Seventeen clinicians who had either offered usual written consent only (n = 6) or both the two-stage pathway (with oral assent before the birth and written consent after the birth) and usual written consent (n = 11) were interviewed. Six themes were identified: (1) team approach to offering participation; (2) consent form as a record; (3) consent and participation as a continual process; (4) different consent pathways for different trials; (5) balance between time, information, and understanding; and (6) validity of consent. Overall, clinicians were supportive of the two-stage consent pathway. Some clinicians felt that in time-critical situations oral assent presented an advantage over the usual written consent as they provided information on a “need to know” basis. However, there was some concern about how much information should be given for oral assent, and how this is understood by women when birth is imminent.

**Conclusions:**

The two-stage pathway for consent developed for use in the Cord Pilot Trial when birth was imminent was acceptable to clinicians for comparable low-risk studies, although some concerns were raised about the practicalities of obtaining oral assent.

**Trial registration:**

ISRCTN Registry, ISRCTN21456601. Registered on 28 February 2013.

**Electronic supplementary material:**

The online version of this article (doi:10.1186/s13063-017-1940-5) contains supplementary material, which is available to authorized users.

## Background

Recruitment to trials when very preterm birth (before 32 weeks gestation) is imminent requires approaching women and their partners at a particularly difficult and stressful time, often with limited time available for them to make an informed decision. This raises challenges for obtaining valid informed consent, both in terms of the practicalities of giving detailed information in a short period of time [1, 2] and in terms of the capacity of parents to absorb and understand the information and to make a voluntary decision [3]. Nevertheless, parents want the opportunity to participate (or allow their infant to participate) in perinatal research and value their role as decision-makers [1, 3–5]. If strategies for offering rapid consent are not developed, women who are at highest risk would be excluded from intrapartum trials, and trials of emergency perinatal or neonatal care would not be possible.

The Cord Pilot Trial compared alternative policies for timing of cord clamping at very preterm birth [6] at eight UK sites. The two arms of the Cord Pilot Trial were: (1) immediate cord clamping, which meant clamping the cord within 20 seconds; and (2) deferred cord clamping, which meant clamping the cord after at least 2 minutes. For births with cord clamping after at least 2 minutes, initial neonatal care was at the bedside. In some cases preterm birth may not happen suddenly. However, in most cases, very preterm birth can be rapid and unexpected, allowing little time for the usual consent process. Therefore a two-stage pathway for consent was developed for use when birth was imminent [7]. The aim was to ensure women and their babies at high risk, who had largely been excluded from previous trials, had the opportunity to participate. This two-stage pathway involved giving a brief description of the trial and offering the woman participation [7]; if she agreed to participate she was randomised and then approached after the birth to give more detailed information and offered written consent for participation in follow up. Of the 261 women randomised, 69 (26%) were recruited following oral assent. None of the women recruited via the two stage-consent pathway who gave oral assent went on to refuse follow up at the written consent stage. The two-stage pathway was developed in discussion with, and was endorsed by, parent representatives from Bliss, the special care baby charity, and the National Childbirth Trust. It is also in line with guidance on valid consent for research while in labour [8]. The aim of this study was to assess clinicians’ views and experiences of offering these two consent pathways. Women’s views and experiences are reported elsewhere (Sawyer, Chhoa, Ayers, Pushpa-Rajah, Duley: Women’s views and experiences of two alternative consent pathways for participation in a preterm intrapartum trial: a qualitative study, submitted).

## Methods

### Participants and procedure

Principal Investigators at the eight sites were contacted between September and December 2014 and asked to provide a list of clinicians who had been involved in offering consent to the Cord Pilot Trial and were willing to be interviewed. Information about the study and an invitation to participate was sent by email to each clinician. The clinician’s positive reply to this email was considered as their agreement to participate in the study. They were then contacted and an interview date arranged.

The interview schedule (Additional file 1) consisted of open-ended questions, which were used to explore clinicians’ views and experiences of inviting women to take part in the trial [5]. Probes were used to explore responses in more depth. Interviews were carried out by a female research assistant (CC) trained in qualitative methods (see Additional file 2 for reporting guidelines for qualitative studies). The interviewer would introduce herself and explain the purpose of the research. Interviews were conducted either in a private hospital room or by telephone, and lasted approximately 20–30 minutes. No one was present apart from the interviewer. Interviews were audio-recorded and transcribed, with all identifying information removed. Data collection ended when data saturation had been achieved (i.e. no new insights were observed).

### Data analysis

Qualitative analysis of the transcripts used inductive thematic analysis to identify, describe and analyse themes and patterns within the data [9]. Thematic analysis was used to inductively (from the data) analyse the interviews. After transcripts were read and re-read for data familiarisation, interviews were coded to generate an initial pool of codes (AS). Codes were then collated into potential themes. Themes were reviewed by three authors (AS, CC and SA) in relation to the generated pool of codes and the entire data set. Finally, definitions and names were generated for each theme. NVivo Version 10 software (QSR International Pty Ltd.) was used to manage codes and themes. For this report, direct quotes are coded (participant number; job role; type of consent) to ensure anonymity.

## Results

Invitations to be interviewed were sent to 20 clinicians, and 17 clinicians from 7 hospitals responded: 5 consultant neonatologists, 3 neonatal or paediatric registrars, 5 neonatal nurses, and 4 midwives. Eleven clinicians (65%), including all the consultants and registrars, had offered women both the two-stage pathway and usual written consent. Six (35%) had offered women standard written consent only, and none had offered the two-stage pathway only.

Six major themes were identified in clinicians’ experiences of offering the two consent pathways: (1) team approach to offering participation; (2) consent form as a record; (3) consent and participation as a continual process; (4) different consent pathways for different trials; (5) balance between time, information, and understanding; and (6) validity of consent. Differences were explored between the professions; however, as there were no overall differences between professional groups (i.e. doctor, nurse, midwife) regarding the content of the themes, the results are presented for the whole sample.

### Team approach to offering participation

Over half of the clinicians discussed the importance of a team approach to inviting women to participate in the trial, regardless of which consent pathway was used. Some clinicians mentioned that they used a team approach to explain the trial to women and invite consent. For example, one neonatologist discussed that both he and the research nurse approached women together and then one of them would follow-up:

I think there’s a real advantage in having me and my research nurse work quite closely together. We quite often make a joint approach to somebody and then I leave it with [research nurse] to follow up or indeed the other way around. (16; Consultant Neonatologist; oral and written consent)

Moreover, some clinicians felt that this approach had the advantage of “covering all bases” with respect to providing potential participants with information: “having the same person always delivering the same thing often there’ll be blind spots or ways of approaching that doesn’t work with other people” (1; Consultant Neonatologist; oral and written consent). The team approach was also seen as a more time-efficient method of offering consent for busy clinicians:

When you’re a busy clinician that does take my time. In [name of hospital] we’re very lucky in that in the daytime we have a lot of research staff who take consent, so they’re brilliant. If we’ve approached a woman during the antenatal counselling giving them a leaflet, then that will then be backed up by a research nurse going to them and taking their consent the following day. And I think that system works really, really well. Written consent is generally not a problem, the only con to it is the time. (8; Neonatal Registrar; oral and written consent)

One clinician noted that it was also important to ask the opinions of other staff about whether it was appropriate to approach a woman: “I did ask the midwives if it was reasonable to make an approach” (16; Consultant Neonatologist; oral and written consent). Some clinicians also thought that the most appropriate person to initially provide information about the study was one of the neonatal staff as part of their antenatal counselling and that this should be embedded in the “normal care of ladies having preterm babies” (9; Research Midwife; written consent).

I think the consent process, particularly for Cord, is quite often intertwined with antenatal counselling. So if you’re about to have a preterm baby what will often happen is someone will come to talk to you about prematurity, preterm babies, what they’re going to do during delivery, what’s likely to happen in the next few hours and subsequently. And those conversations overlap a little bit with the Cord trial consent, because some of the conversations are about what they’re going to do when your baby’s born. So it can be quite difficult to separate those out, so if you had someone who is just doing Cord consent but wasn’t involved in the management of premature babies that would potentially feel a bit artificial. (7; Consultant Neonatologist; oral and written consent).

The importance of regular multidisciplinary training for offering participation in the trial was also commented upon by clinicians. This was highlighted as particularly important for the two-stage consent pathway, when there is often little time available and opportunities to offer oral assent are lost if no trained clinicians are available. One difficulty raised by some clinicians was the rotation of registrars, and the need to ensure new staff members were trained in offering consent was highlighted.

### Consent form as a record

Seven clinicians discussed the issue of whether the signed consent form is a record of the consent process or a legal document to prove consent was given. One of these seven clinicians highlighted that a tension existed between the two perspectives:

So you know there’s a tension between the two sort of approaches, is this a water-tight document? Have you mentioned everything possible that you need to mention? Is it comprehensive, detailed enough to evidence the consent process? And then the other approach that says that this is merely a record of something. (1; Consultant Neonatologist; oral and written consent)

Of the clinicians who discussed this, most were concerned that at the point of oral assent there is a not a clear record of the assent process, as the women do not have to sign anything. One clinician raised the concern that “if you didn’t write it down, it didn’t happen” (16; Consultant Neonatologist; oral and written consent). While the women were not asked to sign a consent form when offered participation via the oral assent pathway, clinicians were asked to document this in women’s clinical notes and to note whether or not they agreed to participate. Three clinicians pointed out this may be particularly important if a participant was to question later whether or not they did actually give oral assent.

I think it’s fundamental that you get informed written consent because then it shows that the woman has been able to make her own choice, she’s not being forced to make a choice. And I suppose if she was to turn around and say, well actually I didn’t consent to this, but she’s signed so, you know, there’s proof that she has. (3; Neonatal Nurse; written consent)

A number of clinicians recognised that if the consent form is a record of agreement, then alternative ways of recording oral assent could be explored to mitigate the risk of not having enough evidence that it had taken place. For example, using a microphone to record the conversation, video record the conversation or ensure that another staff member and or/family member is there to witness the conversation. For situations where time is critical, however, none of these may be practical.

Some clinicians raised the point that written consent forms may be perceived as off-putting to potential participants as they are “very legalistic”. Another clinician thought that presenting parents with a document to sign might pressure them to say yes. As such, it was felt that oral assent may be “less threatening to women and also that you’ve not got this piece of paper, oh here’s pen, sign there, all that kind of stuff. That certainly as a process is much easier” (1; Consultant Neonatologist; oral and written consent). Similarly, a senior research midwife (oral and written consent) noted that oral assent may help a woman in labour make a more informed decision as the focus is not on signing a piece of paper, but rather on the conversation.

Finally, another issue raised about the two-stage consent process was the risk of a lack of a standardised approach:

There’s a sheet saying they’ve had verbal assent, or they’ve given a verbal consent, rather, for the trial. But what’s actually discussed within that could be variable and may not be documented, so one person’s verbal consent may be different form someone else’s. Actually even the same person’s may be different according to the situation and the time variable and other things like language barriers. So the exact nature of what’s been said isn’t really recorded in the same way it is with the written information that’s being given out. (7; Consultant Neonatologist; oral and written consent)

### Consent and participation as a continual process

The process of consent for a randomised trial is not simply the act of offering consent but also includes the ongoing process of building rapport with participants and families, giving information, answering questions whether it is before or after written consent is obtained. The predominant view amongst clinicians was that consent to participation in the trial should be a continual process taking place over time rather than just at a single contact: “Consenting isn’t a one-step process, it’s every time you make contact you’re reaffirming that somebody’s still happy for that to take place” (9; Research Midwife; written consent only). In standard written consent, for example, clinicians often reported meeting with women and their partners and explaining the study at least several times before they asked the woman to make a decision about participation. Some clinicians felt that one of the advantages of consent as a more continual process was that speaking to families on more than one occasion might help women understand and retain the information about the trial. One midwife (written consent) also felt that this repeated contact with families helped develop a rapport between them. The same midwife discussed how this extra time with parents can be used to give general information about preterm birth and mother-baby bonding:

The pros are that you’re going to be able to inform a woman and have dialogue with the woman and get to know them. So, for example, if I’ve gone back to see them a few times on the ward, I’ve often done that social call afterwards so that you build up a bit of a rapport with them. (4; Research Midwife; written consent only)

… if you get written consent and it’s not done in a hurry like oral, loads of loads of opportunity to just go and to chat and to talk about stabilisation and to talk about the relationship between mother and baby. (4; Research Midwife; written consent only)

Although recognised as an optimal method of offering consent it was recognised by some clinicians that the time spent with families in the consent process can be particularly time-consuming.

I do find that it takes an awful long time, it’s not news to spend an hour or more talking to them. I have spent three hours with a family, just because they’ve got so many questions and there’s so much else going on. Not all in one go but over a period of a few days, it’s amounted to at least three hours’ time that you’ve spent within that consent process. (11; Neonatal Nurse; oral and written consent)

One clinician thought that written informed consent is more aligned with consent as a continual process compared to oral assent:

It [consent] should be a process, an on-going process, continuing to re-confirm engagement with the trial and their willingness to continue. I think consent fits better with that and I think it is certainly easier to evidence that you have that process. (16; Consultant Neonatologist; oral and written consent)

The notion of the continuity of the consent process was also prevalent in clinicians’ discussions of oral assent. Provision of information in the first stage of the two-stage consent pathway is on a “need to know”’ basis and women and their partners are given only the minimum information necessary for participation. The majority of clinicians who had used the two-stage pathway for consent thought the minimum information necessary for oral assent were randomisation, the intervention and its risks and the women’s right to decline participation or withdraw from the study.

I find that that with the women, all of the questions that you get are all about the intervention, and randomisation and safety. None of them asks questions about follow up. So I think talking to them about the randomisation is fine and I think if you started to talk to them about all of the follow-up and that kind of thing at that point, you know, I don’t know how much interest they would take in that. Obviously, when you go to them afterwards to ask for consent, that’s when you talk about all that kind of thing. (12; Neonatal Nurse; oral and written consent)

Therefore, the majority of clinicians thought that the written stage in the two-stage pathway was essential in confirming their continuing consent, and in giving the women further information with respect to follow up, the use of their data, etc.

Well I think generally in any study you have to remind people and make sure that they fully understand, but also the specifics because if the woman is in labour or delivery is threatened early I still feel that they are not in the full frame of mind to grasp everything that you’re saying. So being able to come back when they are little bit more relaxed hopefully and can take in more information and not in pain, then yes it’s definitely important. Just reconfirming understanding and that they still wish to be part of the trial. (5; Neonatal Registrar; oral and written consent)

I think it’s important to continue the- essentially you’re starting a conversation with somebody who might be in relatively advanced labour, so it’s about checking understanding as much as anything. And obviously we’re getting someone to sign the dotted line and in doing so you’re kind of cover oneself and getting permission to use information. So really it’s a continuation of a consent conversation and that’s the way it ought to be. (16; Consultant Neonatologist; oral and written consent)

### Different consent pathways for different trials

This theme describes how the two different types of consent processes were viewed as more or less acceptable depending on the type of intervention in the trial. The consensus was that the usual written informed consent process was the optimal form of consent, and should be used wherever possible. Many clinicians thought that the two-stage consent pathway would be appropriate for trials where the intervention is low risk and/or non-complex and thus more acceptable to participants. For example, some clinicians thought that oral assent would be useful in the Total Body Hypothermia (TOBY) trial in which infants had to be randomised within 6 hours of birth [10]. Most clinicians thought the two-stage consent pathway was acceptable for the Cord Pilot Trial, which was essentially viewed as evaluating a more physiological approach than current standard care: “So certainly for a trial like ours where it’s straightforward, then I think it’s really very successful” (1; Consultant Neonatologist; oral and written consent). In comparison, for trials with a higher risk profile, such as certain pharmacological interventions, the two-stage consent pathway was not thought to be appropriate.

The two-stage consent pathway was also thought to be appropriate for trials where a decision about participation needs to be made quickly, for example, emergency interventions/treatment:

I think it depends on the nature of the trial. I don’t think it should be the default option, I think it’s more for emergency or time-critical situations. In that situation I think it’s a reasonable model. (7; Consultant Neonatologist; oral and written consent).

In addition, most clinicians thought oral assent minimised any unnecessary burden to a woman who might already be in considerable distress. A further important advantage of having a two-stage consent pathway for perinatal trials where there is limited time to make a decision was that more women were offered the opportunity to participate in the trial:

I think it would be to capture more people, because the nature of this particular trial is that many women will come just before delivery and there will not be time. If you’re waiting for the ideal time to get written consent for everybody, for this particular trial, it may not happen and you’ll have less people. (5; Neonatal Registrar; oral and written consent)

… it means that you don’t miss patients that would potentially- well, a third of our recruitment would be missed if it hadn’t been there. Because there’s not any risks to what we’re doing, then it would be a shame to miss them if it’s something that they would have liked to have been involved in. (11; Neonatal Nurse; oral and written consent)

Giving more women the opportunity to participate may therefore mean that the final sample is more generalisable, as the higher risk mothers and babies are now included:

The pros are that you can get ladies when they’re close to delivery and still get them into the trial. I think that’s important because we want a good comparison between the two groups. We want the data that we get for them trial to be generalisable, don’t want it to only be applicable to ladies who’ve got plenty of time and you’re doing a section. We want it to be important for babies who are being born prematurely as well. (6; Consultant Neonatologist; oral and written consent)

I think you have to pursue it definitely, because otherwise you’re going to end up with different patient groups and your trial is not going to be valid, your outcome’s not going to be valid. (8; Neonatal Registrar; oral and written consent)

Also, one clinician thought these women, who are eligible but do not have much time, have the right to be offered participation in the study:

And I think they have a right to be offered that and make a decision if we can actually get the information across in a reasonable way to them. (7; Consultant Neonatologist; oral and written consent)

Thus, having both consent pathways available meant that opportunities for recruitment to the trial are not lost where there is limited time for standard written consent procedures and with regards to the Cord Pilot Trial one clinician said that written consent would not have been good on its own.

### Balance between time, information, and understanding

When offering participation in a trial there has to be a balance between the time available and the amount of information given. Oral assent was offered to women when birth was imminent and there was limited time, so many of the women were in labour, in pain or being prepared for a caesarean section. Therefore, information giving and offering consent is happening in an already stressful context. One consultant neonatologist (oral and written consent) mentioned that they often only have time to give the headlines of what the study is about, but also felt that less information was easier for women to grasp in such situations:

… if it’s the headlines then they get their head around a few salient points, then they’re more likely to take that on board then be able to say yes or no, rather than ‘actually there’s too much, I can’t actually think about that now’. (7; Consultant Neonatologist; oral and written consent)

Some clinicians were concerned about how much women in those situations understood and absorbed about the study:

But it is, you know, oral assent is questionable exactly how much information can be given and how much can they actually understand. (3; Research Midwife; written consent only)

… but I must say it is a situation where they are in labour, they are having the stress of having a premature delivery, so you could always argue how much of them are really, just like the woman I mentioned who couldn’t make the decision. I think it’s a difficult time to make a decision that you feel 100% confident with because there are other things on your mind. (5; Neonatal Registrar; oral and written consent)

Most of the clinicians described situations when a woman agreed to the study but after birth had forgotten details about the study or did not have a thorough understanding of the trial, and that this applied to both women who gave oral assent and those who gave standard written consent:

She was happy to take part and was consented and randomised into of the trial into the deferred clamping, but we did need to revisit that afterwards to go through things because although her partner had a reasonably clear recollection, mum was really quite patchy on things. (7; Consultant Neonatologist; oral and written consent)

However, there was general consensus amongst clinicians that the time available varied greatly between women and was just often a function of the situation. For example, two clinicians mentioned that women labour differently and this can affect their focus and understanding. Also, the time available can vary significantly between women when oral assent is offered:

I would think that varies so much in each situation. I don’t think can be general about that, because, like I say, some have seconds to make the decision and some have ten minutes to make the decision, but I think there’s a massive difference in that. (10; Research Nurse; oral and written consent)

One clinician mentioned that for families with lower levels of education, the two-stage consent pathway may not be appropriate as there is often too little time to ensure proper understanding of the trial and what participation involves. It was also mentioned that it was really important, when offering oral assent, that women were approached at the right time and given the right amount of information. As oral assent was being offered at a stressful time, giving lots of information is not appropriate either, as one clinician mentioned: “I think if you try to double the information you might well half the recollection” (7; Consultant Neonatologist; oral and written consent).

### Validity of consent

Fourteen clinicians discussed the issue of validity of women’s consent when offering participation using the two-stage consent pathway. Several expressed concern that in time-critical situations when birth is imminent and women are stressed out and may be in pain, it is questionable whether information about the trial is taken in:

I don’t think it’s as good as written consent lots of time because I don’t think they take in as much information. You could be in a situation where you wonder really have they been properly informed, because if you ask them they’ll say yes they know what’s going on but if you ask afterwards the recollection of what was said is hazy. More so for the mums than the dads, but that’s understandable because the mums are in labour. (7; Consultant Neonatologist; oral and written consent)

The cons are how do you know that that woman who is labour is taking in anything at all what you’re saying to her and would she just agree to anything at that point because she’d rather just get on with having her baby than talking to you. (8; Neonatal Registrar; oral and written consent)

In comparison, four clinicians commented that oral assent might enhance the validity of consent in stressful and difficult circumstances:

… where you’ve got a lady really isn’t in the right frame of mind, she’ll just sign anything. Whereas if you’re actually talking to her and asking her, I think you’ve got more chance of her actually making an informed decision. (9; Research Midwife; written consent only)

Well, if they’re in pain I would be more tempted to go for verbal in that situation rather than written. That’s why I’d go for verbal. I think that’s the way I would go, because it’s quick, you can have a quick overview in between contractions rather than them having to read. You know, so you can say “you can say no”. So I think I would rather go down the road of verbal for that one, and then get the retrospective written. (13; Midwife; oral and written consent)

Several clinicians thought it would be a good idea if the study information was made available to women as early as possible. For example, information about the trial could be given to all women during antenatal visits with community midwives. This would mean that eligible women might have an understanding of the trial when they are first approached about the study by a member of the research team. This would be particularly beneficial for time-critical situations where oral assent is offered.

Because I have been to one or two where I’ve sort of mentioned the trial and they have said to me, oh I think I’ve read about that. They just pick them up at the maternity base where information leaflets are and they’ve said to me, oh I have read something about the Cord trial, which is good so they have a little bit of an insight before they even start. (2; Neonatal Nurse; written consent)

It would probably be a good idea if it wasn’t something they are hearing for the first time and it wouldn’t have sounded experimental to them if they had seen some information about it. So I think that would probably have made it more, maybe, normalised it a bit more. (5; Neonatal Registrar; oral and written consent)

Finally, one clinician thought that “truly informed consent” only happens when the women have time to read the information sheet, ask questions, and have time to think without feeling pressured.

## Discussion

The aim of this study was to explore clinicians’ views and experiences of using two consent pathways during a trial of timing of cord clamping at very preterm birth. Women’s views are reported elsewhere. Overall, the clinicians in our study were supportive of the two-stage consent pathway developed to allow women about to give birth the opportunity to participate in this trial. Some clinicians felt that in this situation oral assent presented an advantage over the usual written consent as they provided information on a “need to know”’ basis, so only minimal information is provided about the study at time of recruitment, with additional information and discussion after the birth. There was some concern about how much information should be given for oral assent, and how this is understood by women when birth is imminent. Others thought that this was a more appropriate approach than putting a piece of paper with lengthy detailed information and legalistic terms in front of a woman when birth was imminent. Clinicians were supportive of a two-stage consent pathway for similar trials with low-risk intervention, but cautious about its appropriateness for higher-risk trials.

Although the clinician completed an oral assent form, the woman was not asked to sign anything at the time of recruitment via the oral assent pathway. This was a concern for some clinicians. Some women in our study said they had forgotten that they agreed to the research, though it is important to note that this was also evident in women recruited using written consent. Documentation from the participant themselves at time of assent may be particularly important if a participant was to question later whether or not they did actually give oral assent. Suggestions for alternative ways of documenting that oral assent have been offered, included audio or video-recording the conversation, and ensuring that another family member is present as a witness. However, these have practical limitations as recording would also require consent and the equipment to be rapidly available, and finding a family member may take time. When birth is imminent or in comparable situations, these would not be possible.

In this study, clinicians considered consent to be a continuous process, rather than a single contact, regardless of which consent pathway was used. Some considered the two-stage consent pathway offered an advantage, as oral assent was followed up with more information and discussion after the birth before the woman was asked for written consent. Continuous consent is an approach recommended by Allmark and Mason [10], where there is initial agreement to participate but there is also continued discussion and information after the treatment. A qualitative study evaluating the effectiveness of continuous consent reported that the validity of consent improved when discussion continues after treatment. However, although parents in the study were positive overall about continuous consent some parents expressed concerns. Specifically, they were worried about receiving information at a later date which could have impacted their original decision [10]. There was agreement that regular training for offering consent to the trial was important. For the two-stage consent pathway, recruitment was predominantly by CORD-trained clinical staff involved in the patient’s direct care, such as doctors and midwives, as research staff (e.g. research midwife or research nurse) were unlikely to be available. If trained clinical and research staff were not available, oral assent could not be offered and so women were not offered the opportunity to participate. Another concern expressed by clinicians was that the information given for oral assent might have been variable. One way to monitor variability would be for clinicians to be provided with a structured format for writing down their conversations for oral assent.

The Cord Pilot Trial compared interventions during birth, and recruitment to the trial was often when women were in labour and/or when birth was imminent. The clinicians in our study were concerned about how much information is understood by women when birth is imminent, particularly with intrapartum women who may be in pain or having a consent conversation in between contractions. For this reason, some clinicians felt that the two-stage consent pathway with only minimal information about the trial provided at recruitment is adequate, and a more appropriate approach than the usual written consent process. Previous research has found that parents agree with this, valuing information about potential risks and benefits over details of the study procedures [11, 12]. Women in our study who were recruited when birth was imminent and there was little time to make a decision also felt it was easier to consider participation when clinicians provided minimal and clear information about the trial. This suggests that consent in time-critical situations could focus on information that is relevant to the immediate situation, leaving other issues such as discussion of follow up and signing the consent form until after the birth. Moreover, consent forms and signatures do not ensure informed decision-making [13] and some clinicians in our study agreed, pointing out that in an emergency or stressful situation women may just sign the consent form in order to get the researcher to go away. Indeed, previous research suggests that parents may be more likely to engage in a conversation about a study when the consent process conversation is not focused around a consent form [14] and clinicians in our study agreed with this. This has implications for the validity of consent. The consent form is less important than communicating information in a manner that is effective and appropriate given the situation, and for some situations oral assent may provide the best approach for doing so.

Researchers have questioned the capacity of parents to absorb and understand information and make a fully informed decision in emergency situations [3] but women and parents want the opportunity to participate and to make their own decisions [5] and a great deal of importance has been attached by codes of ethics to gaining informed consent for medical research [15]. If the problem of rapid parental consent cannot be successfully addressed, there is a risk that some valuable research in the areas of preterm birth and emergency perinatal care will not be possible. The two-stage consent pathway used in the Cord Pilot Trial is one strategy with potential to overcome some of the problems of gaining consent for neonatal and perinatal research when there is insufficient time for the usual single-stage informed consent process. The importance of offering participation in trials when time is critical is also reflected in the two-stage consent pathway developed for the Cord Pilot Trial being incorporated into the Royal College of Obstetricians and Gynaecologists clinical governance advice [16]. The two-stage pathway could also be used in clinical scenarios where standard written consent is difficult (e.g. non-planned surgery where urgent medical intervention is needed) and has potential to overcome challenges of obtaining consent in the standard way for non-planned urgent interventions.

However, recent systematic reviews of the literature on consent in neonatal and perinatal research have highlighted a significant gap in the empirical and theoretical literature of any detailed discussion of a process of emergency assent followed later by full consent [5, 17]. Some concern has been raised that consent at the second stage only provides permission to continue and cannot provide retrospective consent. This is particularly relevant to one-off interventions; although consent would still be applicable to use of personal data for research and subsequent follow up. Therefore, considered theoretical and empirical attention to this process is urgently needed, where parents assent or refuse participation during the emergency and later give full consent to continue participation and follow up.

### Strengths and limitations

A recent review of ethics issues around consent in clinical trials with preterm or sick neonates identified a significant gap in the literature evaluating emergency assent [5]. This is the first study to explore clinicians’ experiences of inviting women to participate in a randomised trial using a two-stage oral assent pathway, with initial oral assent followed by written consent. The use of detailed qualitative methods allowed an in-depth exploration of these experiences and allows a comparison of the two consent pathways. Clinical staff members with a range of clinical backgrounds from seven of the eight UK hospitals who participated in the Cord Pilot Trial were interviewed, suggesting that the findings are representative of experiences in this trial. Our results are based on a single trial, and other factors may be more important, or less important, in trials with different populations and different risk and benefit profiles.

## Conclusions

Improving access to emergency trials when time is critical is important if we are to develop an appropriate evidence base for these clinical problems. The two-stage consent pathway developed for use in the Cord Pilot Trial when birth was imminent was acceptable to clinicians for comparable low-risk studies, although some concerns were raised about the practicalities of obtaining oral assent. This is the first empirical study to evaluate a novel process of seeking parental consent for neonatal and perinatal research when there is insufficient time for the usual single-stage informed consent process. The process requires further development and evaluation including evaluating this process in trials with different populations and different risk and benefit profiles. Different ways of documenting assent should also be explored to enable a record of the process. However, the option of oral assent followed later by full consent should be considered for similar trials where the intervention is low risk and time is critical.

## Additional files

Additional file 1: Interview schedule (DOC 33 kb)

Additional file 2: Reporting guidelines for qualitative studies (DOCX 15 kb)
