# Supplementary material for: Clinicians’ views and experiences of offering two alternative consent pathways for participation in a preterm intrapartum trial: a qualitative study
Source: Trials. 2017 Apr 26;18:196. doi: 10.1186/s13063-017-1940-5 (PMC5406899; doi:10.1186/s13063-017-1940-5)
Supplement: Supplementary file 1 — Interview schedule (DOC 33 kb) [file 13063_2017_1940_MOESM1_ESM.doc]

**Appendix A: Interview Schedule**

**Job title:**

**Length of time in role:**

**Opening questions:**

- What is your experience of explaining randomised trials to potential participants, and asking for consent?
- Did you receive any training about asking for consent to the Cord Pilot Trial?
- If yes, were you happy with this training?
  - If you were not happy with the training, what was missing?
- Can you tell me about your experiences of inviting women to participate in the Cord Pilot Trial?
  - Approximately, what was the timeframe of your involvement in the Cord Pilot Trial?
  - Did you ask for written consent and/or oral assent?
  - In your experience of asking for consent to the Cord Pilot Trial, did women agree or decline to take part in the trial?
- Do you think women saw the information about the Cord Pilot Trial available to them in the clinics?
- Do you think this information was useful?
- Is there another/better way we could give women initial information?

**Written informed consent**

- What is your experience of asking people to give written informed consent for trials?
- For you, what are the relative pros and cons of asking people for written informed consent before the birth?
- Was there anything that could have been done differently to improve it?
- At the time of written consent:
- Did you feel women were given enough information
  - If no – what was missing?
- Did you think they had enough time to make the decision?
  - If no, please explain
- Did you feel that this method (asking for consent prior to the birth) was an adequate way of asking for informed consent?

**Oral assent (verbal consent) followed by written consent**

What part of the two-stage consent process were you involved in: asking for oral assent, written consent, or both parts?

*Ask the following questions if participant asked for oral assent or both parts*

- What is your experience with using oral assent for consent?
- For you, what are the relative pros and cons of oral assent (verbal consent)?
- Was there anything that could have been done differently to improve it?
- At the time of oral assent:
- Did you feel women were given enough information
  - If no – what was missing?
- Did you think they had enough time to make the decision?
  - If no, please explain

*Ask the following questions if participant asked for written consent or both parts*

- At the time of written consent afterwards:
- Did you feel women were given enough information
  - If no – what was missing?
- Did you think they had enough time to make the decision?
  - If no, please explain
- Did you feel that this method (asking for written consent after the birth) was an adequate method of asking for consent?

*Question for all the participants involved in the two-stage process*

- Did you get any feedback from the women about either oral assent or being asked for written consent afterwards?
- If yes, what was the feedback?
- Would you recommend this 2-stage process for other trials? If yes what sorts of trials?
